# Sarcopenia Is Associated With Hematologic Toxicity During Chemoradiotherapy in Patients With Anal Carcinoma

**DOI:** 10.3389/fonc.2020.01576

**Published:** 2020-08-12

**Authors:** Daniel Martin, Jens von der Grün, Claus Rödel, Emmanouil Fokas

**Affiliations:** ^1^Department of Radiotherapy and Oncology, University Hospital, Goethe University, Frankfurt, Germany; ^2^Frankfurt Cancer Institute, Frankfurt, Germany; ^3^German Cancer Research Center (DKFZ), Heidelberg, Germany; ^4^German Cancer Consortium (DKTK), Frankfurt, Germany

**Keywords:** anal cancer, toxicity, outcome, sarcopenia, leukopenia, thrombopenia

## Abstract

**Purpose:**

Sarcopenia, defined as a loss of muscle mass and quality, has been associated with impaired oncological outcome and treatment toxicities in several malignancies. However, its role in anal squamous cell carcinoma (ASCC) remains less well explored.

**Methods/Materials:**

Planning CT scans were used to measure cross-sectional skeletal muscle area (SMA) to calculate the skeletal muscle index (SMI). The association of sarcopenia with clinical and treatment-related parameters, and toxicity was assessed in 114 patients with ASCC that underwent standard 5-Fluorouracil/Mitomycin C chemoradiotherapy (CRT). The prognostic impact of sarcopenia on local relapse-free survival (LRFS), disease-free survival (DFS), and overall survival was examined using a Cox regression analysis.

**Results:**

29 (25.4%) patients had sarcopenia. Patients with sarcopenia had lower baseline hemoglobin levels (*p* = 0.002), worse Karnofsky Performance Status (*p* = 0.001) lower BMI (*p* < 0.001), and a significantly lower body surface area (*p* = 0.03), and lower incidence of involved lymph nodes (*p* = 0.03). Regarding acute toxicity, sarcopenia was associated with a significantly higher incidence of ≥grade 3leukopenia (OR: 3.5; 95% CI: 1.6–7.5, *p* = 0.007) and ≥grade 3 thrombopenia (OR: 5.1; 95% CI: 1.3–21, *p* = 0.018) after CRT. Despite higher hematologic toxicity in sarcopenic patients, total treatment time was similar between patients with and without sarcopenia (median 44 vs 45 days, *p* = 0.95). There was no significant prognostic impact of sarcopenia on either LRFS, DFS, or OS.

**Conclusion:**

This is the largest study to assess the impact of sarcopenia on toxicity and oncological outcome in patients with ASCC. Increased clinician awareness of higher hematological toxicity risk is needed for sarcopenic patients with ASCC undergoing CRT to facilitate closer monitoring of side effects and earlier introduction of supportive measures. Further prospective studies are needed to elucidate the prognostic role and impact of sarcopenia on CRT-related toxicity in ASCC.

## Introduction

Anal squamous cell carcinoma (ASCC) is associated with human papilloma virus (HPV) infection (1). The standard treatment is primary chemoradiotherapy (CRT) and durable remissions can be achieved in a high proportion of patients with early disease (2). Nevertheless, lymph node positivity, or large tumors are associated with significantly worse outcomes (3, 4). Additionally, patient specific factors such as sex (3) and performance status (5) can impact the prognosis of the patients.

Sarcopenia, defined as loss of muscle quantity and muscle strength, has mainly been recognized as an age-related phenomenon, but is also associated with cancer cachexia (6). In the field of oncology, sarcopenia is defined as a loss of muscle quantity measured as a low appendicular skeletal muscle index (SMI), that can be measured by dual energy x ray absorptiometry (6). Most widely used are computed tomography (CT) scans of the third lumbar area to measure the skeletal muscle area (SMA) to calculate the SMI by adjusting for height. This measurement correlates closely to the whole body muscle quantity (7).

Sarcopenia, measured by CT scans can result in adverse clinical outcome in different tumor types, such as lung cancer, or rectal cancer (8–11). In gastrointestinal cancers, the risk of malnutrition and, in consequence, sarcopenia, is higher due to the associated-eating disorder, vomiting, and/or increased metabolic consumption. A meta-analysis of 70 studies in patients with gastrointestinal cancer showed that pre-treatment sarcopenia is associated with adverse oncological outcome and surgical complications (12). In ASCC, a small retrospective analysis in 64 patients showed an adverse prognostic role of sarcopenia for overall survival (13). In the present study we aimed to validate these findings in a cohort of 114 patients with ASCC treated homogenously with primary, standard CRT. Additionally, we wanted to investigate whether markers of inflammation are associated with sarcopenia, as suggested by the literature (14).

## Materials and Methods

### Patients and Treatment

**FIGURE 1:**
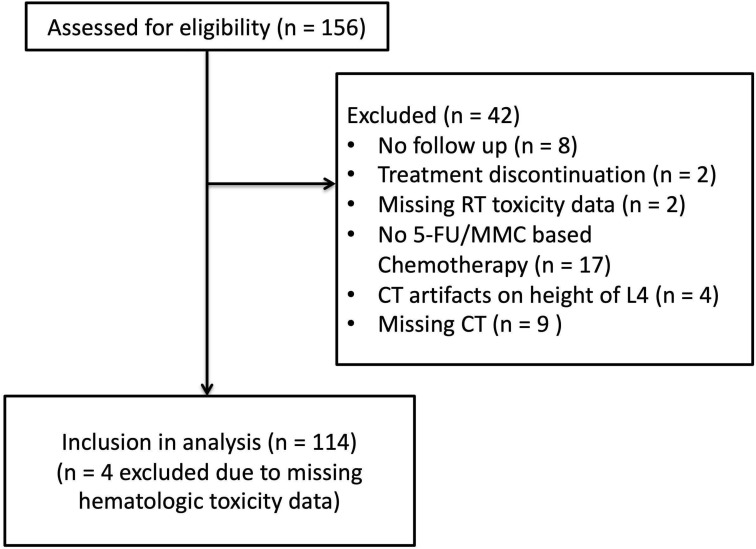
CONSORT diagram showing the selection of patients for study eligibility.

156 patients treated between 2007 and 2018 were assessed for eligibility (Figure 1). After exclusion of patients with missing follow up, missing data, other chemotherapy regimens that 5-fluorouracil (5-FU), and mitomycin C (MMC) or CT artifacts we identified 114 patients treated for localized ASCC and staged according to UICC version 7. All patients were treated with CRT with a planned radiotherapy dose of 50–50.4 Gy in 1.8–2 Gy fractions for the primary tumor and elective lymph nodes with an additional radiotherapy boost to gross tumor of 5.4–9 Gy. Intensity modulated radiotherapy (IMRT) for ASCC was implemented in 2011. Patients treated earlier received 3D-conformal radiotherapy (3DCRT). Concurrent chemotherapy consisted of 5-fluorouracil (5-FU, 1000 mg/m^2^/day, or 800 mg/m^2^/day) as four- or five-day continuous infusion and MMC given as an intravenous bolus (10 mg/m^2^) on day one of each cycle. Concurrent chemotherapy was applied in the first and fifth week of CRT. Acute toxicities were assessed weekly as part of the clinical routine using CTCAE (version 3 and 4).

Treatment response was first assessed 8–10 weeks after completion of CRT. Afterward, routine follow-up was performed every 3 months for the first 2 years and 6-month intervals afterward. Routine follow-up examinations included physical and digital rectal examination, proctoscopy, and pelvic imaging. Written consent and approval from the institutional review board was obtained for the present analysis.

### CT Based Measurement of Sarcopenia

Planning-CT scans were obtained in supine position from all patients using a Brilliance^TM^ Big Bore Scanner (Philips, Netherlands). Scan parameters were as follows: slice thickness 3 mm, tube voltage 120 kv, FOV 600 mm, and tube current from 250–400 mA. No contrast agents were used. A common landmark for CT based sarcopenia measurement is the third lumbar vertebra (L3). As not all available CT scans included L3, we used L4 as a landmark and used established cut-offs that were derived from a cohort of 735 healthy potential kidney donors (15). A single image from the most inferior aspect of L4 was exported. SMA and SMI were measured using ImageJ (Version 2.0.0-rc-69/1.52p) as described elsewhere in detail (16). In short, the outer and inner abdominal musculature was contoured and both areas were measured, thresholded for skeletal musculature [-29 Hounsfield units (HU) to 150 HU] and subtracted from each other to calculate the SMA at this slice. SMA was then divided by the square of the patient’s height to derive the SMI using the following formula:

$$S\,M\,I=\frac{O\,u\,t\,e\,r\,m\,u\,s\,c\,u\,l\,a\,t\,u\,r\,e\,a\,r\,e\,a-I\,n\,n\,e\,r\,m\,u\,s\,c\,u\,l\,a\,t\,u\,r\,e\,a\,r\,e\,a}{H\,e\,i\,g\,h\,t^{2}}$$

(see Figure 2A).

**FIGURE 2:**
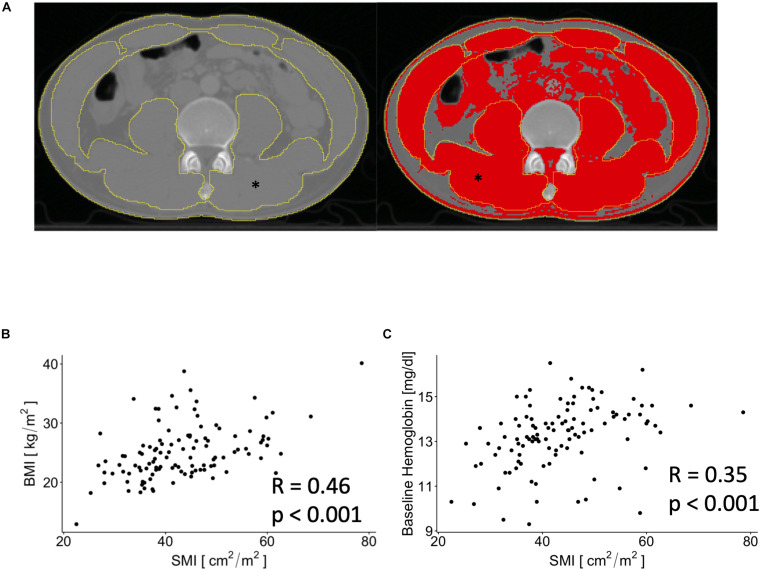
Exemplary image that show contouring of outer and inner musculature area before (left) and after (right) thresholding for skeletal muscle associated Hounsfield units. ^∗^depicts the skeletal muscle area (SMA) on both images. **(A)** Scatterplots showing associations between SMI and body mass index (BMI) and baseline hemoglobin levels **(B,C)**.

Measurements were done by DM. Precision was measured in two ways. Repeat measurements were done in a subset of 25 patients (*r* = 0.998, *p* < 0.001) and one randomly selected patient was measured five consecutive times (SMA: 122 ± 1.33 cm^2^; mean ± SD).

### Statistical Analysis

Differences between groups were assessed using Fisher’s exact test for categorical variables, if not indicated otherwise. For continuous variables, differences between groups were assessed using Wilcoxon signed rank test. Correlations between continuous variables were assessed using the Pearson correlation coefficient or Spearman’s rank correlation depending on applicability. Body surface area (BSA) was calculated using the Mosteller formula (17). Survival times were calculated from start of CRT to the date of respective events or last follow-up. Local relapse-free survival (LRFS) was calculated using non-complete response at first restaging, locoregional recurrence after initial complete response or death of any cause as event. Disease-free survival (DFS) was calculated using the date of diagnosis of locoregional failure, distant metastases, or death of any cause. OS was assessed from the date of diagnosis with death of any cause as the respective event. Differences in survival were calculated using Cox regression analysis. The assumption of proportional hazard was verified by assessing the scaled Schoenfeld residuals. *Z*-scores for SMI were calculated for male and female patients separately because of the significant impact of sex on SMI. These *z*-scores were then used in additional Cox regression analyses. Odds ratios for developing high grade leukopenia or thrombopenia were calculated using logistic regression analysis. All statistical analysis was performed with R (Version 3.5) (18). A *p*-value < 0.05 was considered significant.

## Results

### Patient and Treatment Characteristics

Median age of the patients was 58.5 years; 58 were male, and 56 were female. The median body mass index (BMI) was 24.3 kg/m^2^ and 25 (22%) patients were HIV-positive. The majority (*n* = 72; 63%) of patients had a T1 or T2 ASCC, and 50% of the patients had positive lymph node(s) at initial presentation. All patients were treated using combined CRT; 95 (83%) patients were treated with IMRT to a median total dose of 59.4 Gy. Median follow-up was 30 months.

### Sarcopenia and Clinicopathologic Characteristics

We dichotomized the patients into having sarcopenia or no sarcopenia according to sex and SMI using the established cut-offs at the fourth lumbar vertebrae as previously described, i.e., 41.3 cm^2^/m^2^ for male and 34.2 cm^2^/m^2^ for female patients (15). As such, 29 (25.4%) of patients were scored as having sarcopenia (Table 1). SMI was positively correlated with BMI (*p* < 0.001, Figure 2B) and showed a negative correlation with age (*R* = −0.24, *p* < 0.01). We found a positive correlation between SMI and baseline hemoglobin levels (*p* < 0.001, Figure 2C), whereas no significant association between baseline white blood cell count and SMI was observed (*p* = 0.06).

**TABLE 1:** Patient characteristics and association of sarcopenia with clinicopathologic parameters and hematologic toxicities.

	*N*	Sarcopenia (%)	No Sarcopenia (%)	*p*-value
**Age, years**		59(43−87)^∗^	58(26−83)^∗^	n.s.
**Sex**				
Male	58	15 (52)	43 (51)	
Female	56	14 (48)	42 (49)	n.s.
**T Stage**				
T1-T2	72	16 (55)	56 (66)	
T3-T4	42	13 (45)	29 (34)	n.s.
**N Stage**				
N0	57	20 (69)	37 (44)	
N+	57	9 (31)	48 (56)	**0.03**
**HIV status**				
HIV-negative	89	22 (76)	67 (79)	
HIV-positive	25	7 (24)	18 (21)	n.s.
**KPS**				
100	83	14 (48)	69 (81)	
≥90	31	15 (52)	16 (19)	**0.001**
**BMIkg/m^2^**		21.9(13−34)^∗^	25.6(19−40)^∗^	**<0.001**
**BSA m^2^**		1.80(1.4−2.3)^∗^	1.89(1.5−2.4)^∗^	**0.03**

*BMI, body mass index; HIV, human immunodeficiency virus; IMRT, intensity modulated radiotherapy; KPS, Karnofsky Performance Scale; and ^∗^median (range). Bold values are significant.*

**FIGURE 3:**
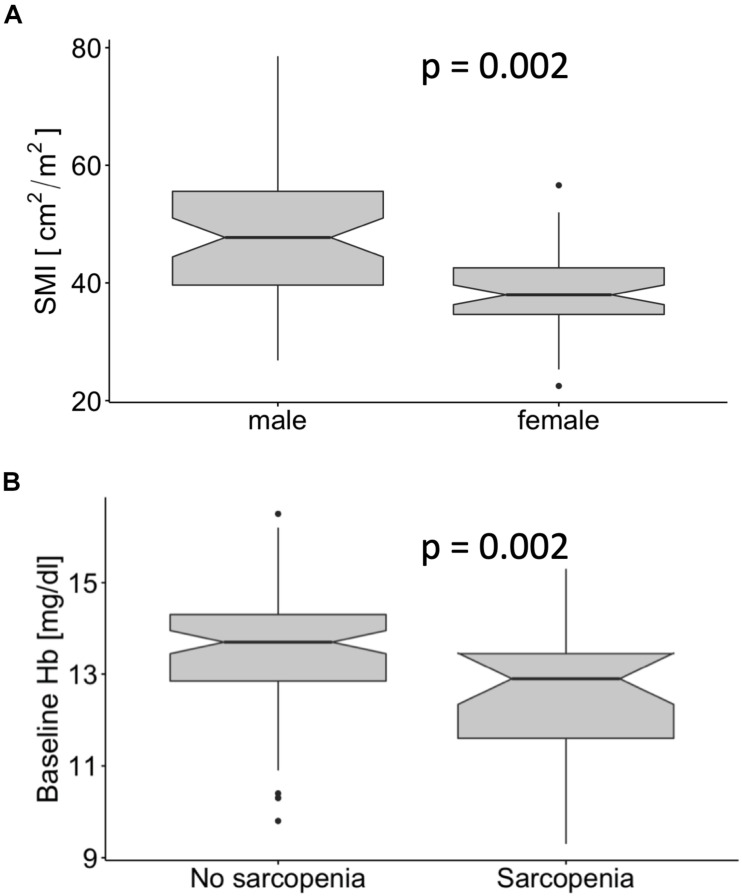
Male patients had a significantly higher skeletal muscle index (SMI) than female patients **(A)**. The baseline hemoglobin levels in patients with sarcopenia were significantly lower **(B)**.

Male patients had a significantly higher SMI [47.7 (39.6–55.5) cm^2^/m^2^ vs. 38.0 (34.7–42.6) cm^2^/m^2^, median, and interquartile range, *p* < 0.001, Figure 3A]. Although male patients had significantly higher baseline hemoglobin levels (*p* < 0.001, data not shown), the use of the sex-specific cut-offs showed that patients with sarcopenia had significantly lower baseline hemoglobin independently of sex (*p* = 0.002, Figure 3B). There was no significant association between the occurrence of sarcopenia and sex, T-Stage or HIV status (Table 1). A higher proportion of patients staged as cN0 had sarcopenia compared to cN + patients (*p* = 0.03). Additionally, a worse Karnofsky Performance Status (KPS; *p* = 0.001), a lower BMI (*p* < 0.001), and a significantly lower BSA (*p* = 0.03, Table 1) were observed in sarcopenic patients (Table 1).

Patients with sarcopenia had a significantly higher incidence of acute CTCAE grade ≥ 3 leukopenia and thrombopenia during CRT (*p* = 0.009 and *p* = 0.025, respectively, Table 2). There was no significant difference between planned and applied dose for 5-FU and MMC, respectively. Additionally, there was no significant difference with regards to any chemotherapy dose reduction (*p* = 0.074, Table 2). The OR for patients with sarcopenia having ≥ grade 3 leukopenia was 3.5 (95% CI: 1.6–7.5, *p* = 0.007) and the OR for developing ≥ grade 3 thrombopenia was 5.1 (95% CI: 1.3–21, *p* = 0.018). Despite more hematologic toxicities in sarcopenic patients, the total treatment time of CRT was similar between patients with and without sarcopenia in our series (median 44 vs 45 days, *p* = 0.95). Also, sarcopenia did not impact on gastrointestinal or skin toxicity (Table 2).

**TABLE 2:** Association of sarcopenia with treatment characteristics and acute toxicity in patients with ASCC after CRT.

	*N*	Sarcopenia (%)	No Sarcopenia (%)	*p*-value
**RT modality**				
3DCRT	19	7(24)	12 (14)	
IMRT	95	22 (76)	73 (86)	n.s.
**Radiotherapy total dose, Gy**		59.4(50.4−59.4)^∗^	59.4(50.4−59.4)^∗^	n.s.
**Chemotherapy**				
5-FU (8000 mg/m^2^ planned)		8000(2000−8000)	8000(3000−8000)	n.s.
MMC (20 mg/m^2^ planned)		20(7−20)	20(7.5−20)	n.s.
**Dose reduction**				
No dose reduction		18 (62)	67 (79)	
Dose reduction		11 (38)	18 (11)	0.074
**Leukopenia (*n* = 110)**				
CTCAE < 3	73	12 (44)	61 (73)	
CTCAE 3/4	37	15 (56)	22 (27)	**0.009**
**Thrombopenia (*n* = 110)**				
CTCAE < 3	99	21 (78)	78 (94)	
CTCAE 3/4	11	6 (22)	5 (6)	**0.025**
**Diarrhea (*n* = 114)**				
CTCAE < 3	106	81 (95)	25 (86)	
CTCAE 3/4	8	4 (5)	4 (14)	n.s.
**Dermatitis (*n* = 114)**				
CTCAE < 3	69	49 (57)	19 (66)	
CTCAE 3/4	46	36 (43)	10 (34)	n.s.

*ASCC, anal squamous cell carcinoma; CTCAE, Common Terminology Criteria for Adverse Events; Gy, Gray; IMRT, intensity modulated radiotherapy; KPS, Karnofsky Performance Scale; RT, radiotherapy; and 3DCRT, 3D-conformal radiotherapy. Note the different total number of patients with available laboratory parameters. ^∗^median (range). Bold values are significant.*

### Sarcopenia and Clinical Outcome

Cox regression analysis was used to determine the prognostic impact of sarcopenia and other established clinicopathologic parameters. The results are summarized in Table 3. We failed to detect a prognostic impact of sarcopenia for either LRFS, DFS, or OS. Patients with advanced T-Stage, N + disease and male sex had significantly worse outcome for all three oncological endpoints (Table 3). The use of bivariate cut-offs for continuous variables in order to estimate outcome can lead to loss of information and to biased results (19) and should optimally be avoided. In order to evaluate the impact of SMI as continuous variable we calculated *z*-scores for for SMI in male and female patients separately and did a cox regression analysis using these scores for all end points and found that SMI had no impact on LRFS DFS or OS (Table 4). In order to reproduce the findings of the above mentioned study in ASCC, we performed a separate analysis using the cut-offs specified in the article (13), but there was no association with any of the survival endpoints (data not shown).

**TABLE 3:** Univariate analysis of prognostic factors regarding LRFS, DFS, and OS.

	HR	95% CI	*p*-value
**Locoregional-relapse free survival**			
T-stage (T3-4 vs. T1-2)	3.06	1.25–7.48	**0.01**
N-stage (N + vs. N0)	4.77	1.59–14.31	**0.002**
Age	0.99	0.96–1.04	n.s.
Sex (female vs. male)	0.30	0.11–0.84	**0.02**
Sarcopenia (yes vs. no)	1.01	0.37–2.79	n.s.
**Disease-free survival**			
T-stage (T3-4 vs. T1-2)	3.5	1.6–7.64	**0.001**
N-stage (N + vs. N0)	4.33	1.74–10.74	**0.002**
Age	1.01	0.98–1.04	n.s.
Sex (female vs. male)	0.32	0.13–0.75	**0.009**
Sarcopenia (yes vs. no)	1.04	0.44–2.46	n.s.
**Overall survival**			
T-stage (T3-4 vs. T1-2)	7	2.24–21.9	**<0.001**
N-stage (N + vs. N0)	6.01	1.69–21.32	**0.006**
Age	1.02	0.97–1.06	n.s.
Sex (female vs. male)	0.15	0.03–0.66	**0.01**
Sarcopenia (yes vs. no)	1.94	0.70–5.33	n.s.

*LRFS, locoregional relapse-free survival; DFS, disease-free survival; OS, overall survival; and n.s. not significant. Bold values are significant.*

**TABLE 4:** Multivariate analysis for SMI using *z*-scores that were calculated for male and female patients separately.

	HR	95% CI	*p*-value
Locoregional-relapse free survival	0.97	0.62–1.52	0.9
Disease-free survival	1.04	0.71–1.53	0.8
Overall survival	0.78	0.48–1.29	0.3

## Discussion

Accumulating evidence indicates an adverse impact of sarcopenia on the prognosis of patients with various malignancies (20), however, in ASCC its role remains less well explored. In the present study, we found that sarcopenia resulted in worse hematological toxicity in patients with ASCC treated with primary CRT. A total of 29 (25.4%) patients in our cohort were scored as having sarcopenia, which is in line with the only other report about sarcopenia in ASCC (13). However, in contrast to this study that reported worse OS in sarcopenic patients, we failed to identify a prognostic role of sarcopenia for either locoregional control, DFS or OS. Additionally, we found no association between treatment outcome and SMI as a continuous variable after adjusting for sex.

Paradoxically, involvement of lymph nodes (cN+) was less common in patients with sarcopenia in our cohort. This is a surprising finding as cN+ status is generally linked to a poor prognosis in ASCC. The study by Bingmer et al. did not report N-stage, although the available AJCC stages suggested a trend toward more advanced stages in non-sarcopenic patients (13). Nevertheless, the reason behind this unexpected finding remains unclear.

We found a positive correlation between SMI and baseline hemoglobin levels. As male patients had significantly higher baseline hemoglobin levels with a higher SMI we additionally analyzed the association between sarcopenia and hemoglobin levels using the sex specific cut-offs, and found that patients with sarcopenia had lower baseline hemoglobin levels independent of sex. This is in line with published data on sarcopenia (21). A link between chronic inflammation and sarcopenia has been proposed (14, 22). Elevated levels of tumor-necrosis factor alpha (TNF-alpha) can lead to activation of the Akt/mTOR pathway, which in turn increases muscle catabolism (23). Another study found a correlation between increased myofibrosis and myosteatosis in human tissue and serum levels of c-reactive protein (CRP) (24). Subsequently, chronic inflammation can lead to anemia through several pathways, e.g., induction of the master regulator of iron homeostasis hepcidin by Interleukin-6 or the inhibition of erythropoietin formation in the kidney mediated by Interleukin-1 and TNF-alpha (25). Nevertheless, we found no correlation between SMI and white blood cell count as a marker of inflammation in our cohort.

Patients with sarcopenia had a higher possibility to develop high grade leukopenia or thrombopenia during CRT, which is in line to previous studies in different malignancies. Sarcopenia was associated with poor chemotherapy tolerance and prolonged treatment breaks in 246 patients with head and neck cancer (26). Another study among various types of cancer also showed higher chemotherapy toxicity in patients with sarcopenia (27), whereas a meta-analysis also revealed higher treatment toxicity in sarcopenic patients with breast cancer (28). Sarcopenia was also associated with more postoperative complications in gastrointestinal cancers (12).

There are no definite pathophysiological explanations for this phenomenon. One possibility for increased hematologic toxicity after CRT could be altered pharmacokinetics in patients with sarcopenia (29, 30). Most chemotherapeutic agents are dosed by BSA without taking body composition into account, which could lead to overdosing. Indeed, a study in patients that were treated with 5-FU and leucovorin for stage II/III colon cancer revealed that a low lean body mass predicted toxicity when 5-FU is dosed per BSA (31). Patients who received more than 20 mg/kg 5-FU per kg lean body mass did not differ in BSA or BMI but had a significantly lower SMI (31). The impact of dose per lean body mass on treatment toxicity of chemotherapy regimens has been subsequently reported in two other cohorts of colorectal cancer patients and in non-small cell lung cancer (32–35). Similar findings were revealed for capecitabine toxicity in breast cancer patients using established sarcopenia measurements instead of lean body mass (36). Mechanistically, another study showed that pharmacokinetic clearance of epirubicin chemotherapy was significantly associated with lean body mass in patients with breast cancer (37). Despite the increased hematological toxicity after CRT sarcopenia did not lead to treatment interruptions in our cohort, which is encouraging as prolonged treatment time can lead to impaired outcome (38).

Our study has limitations. First, we used a different landmark for measurement of SMI compared to other studies that have assessed sarcopenia in cancer patients, although our measurements and cut-offs are supported by a large study in healthy potential kidney donors (15). Secondly, this study constitutes a retrospective analysis that can lead to selection bias.

In conclusion, this is, to the best of our knowledge, the largest study in ASCC patients treated with CRT to assess the role of sarcopenia. Despite the lack of prognostic value, sarcopenia was associated with higher incidence of hematologic toxicity during CRT. Our findings have implications in the clinical setting as increased clinician awareness of higher CRT toxicity risk in sarcopenic patients with ASCC is needed to facilitate closer monitoring of side effects and earlier introduction of supportive measures.

## Data Availability Statement

The raw data supporting the conclusions of this article will be made available by the authors, without undue reservation.

## Ethics Statement

The studies involving human participants were reviewed and approved by Ethik-Kommission des Universitätsklinikums der Goethe-Universität. Written informed consent for participation was not required for this study in accordance with the national legislation and the institutional requirements.

## Author Contributions

DM, CR, and EF contributed to conception and design of the study. DM and JG organized the database. DM and EF performed the statistical analysis. DM wrote the first draft of the manuscript. DM, JG, EF, and CR wrote sections of the manuscript. All authors contributed to manuscript revision, read, and approved the submitted version.

## Conflict of Interest

The authors declare that the research was conducted in the absence of any commercial or financial relationships that could be construed as a potential conflict of interest.
